# Characterization of the wheat cultivars against *Tilletia controversa* Kühn, causal agent of wheat dwarf bunt

**DOI:** 10.1038/s41598-020-65748-w

**Published:** 2020-06-03

**Authors:** Ghulam Muhae-Ud-Din, Delai Chen, Taiguo Liu, Wanquan Chen, Li Gao

**Affiliations:** 1 State Key Laboratory for Biology of Plant Disease and Insect Pests, Institute of Plant Protection, Chinese Academy of Agricultural Sciences, Beijing, 100193 China; 2 College of Plant Protection, Gansu Agricultural University, Lanzhou, Gansu 730070 China

**Keywords:** Developmental biology, Microbiology, Plant sciences

## Abstract

Wheat is one of the most important staple crops. *Tilletia controversa* Kühn is the causal agent of wheat dwarf bunt. In this study, a resistant wheat cultivar displayed significantly higher expression of pathogenesis-related genes than a susceptible cultivar at 7 days post inoculation (DPI) with *T. controversa*. Similarly, the expression was high in the resistant cultivar after exogenous application of phytohormones, including salicylic acid. The expression of pathogenesis-related genes, especially chitinase 4, was high in the resistant cultivar, while LPT-1 was down regulated after *T. controversa* infection. Callose deposition was greater in the resistant cultivar than in the susceptible cultivar at 10 DPI. Confocal microscopy was used to track the fungal hyphae in both cultivars in anther and ovary cells. The anthers and ovaries of the susceptible cultivar were infected by *T. controversa* at 7 and 15 DPI. There were no fungal hyphae in anther and ovary cells in the resistant cultivar until 10 and 23 DPI, respectively. Moreover, anther length and width were negatively influenced by *T. controversa* at 16 DPI. The plant height was also affected by fungal infection. Ultimately, resistance to *T. controversa* was achieved in cultivars via the regulation of the expression of defense-related and pathogenesis-related genes.

## Introduction

Wheat (*Triticum aestivum* L.) is one of the most important staple food crops in most agricultural regions^1,2^. Plant diseases affect wheat crops, decreasing productivity worldwide and severely compromising food security^3^. Dwarf bunt is a soilborne and seedborne disease in many areas of the world^4,5^ and is caused by *Tilletia controversa* Kühn^6,7^. The teliospores or bunt sori of *T. controversa* can last for 10 years in soil and are easily dispersed from field to field with soil or can be taken to new places by infected wheat seeds^8^. These bunt sori replace the grain material with brown-black teliospores with a strong odor like that of rotting fish^9^, which heavily degrades the quality of the wheat seeds and flour. Even slightly low infection levels can result in a noticeable smell in flour minced from infected grains^10,11^. Additionally, *T. controversa* is an internationally important quarantine pathogen, and presently, many countries have strict restrictions on importing wheat from areas where the disease occurs^8,12^.

In flowering plants, the male reproductive organ, the stamen, has four anther lobes, each having a microsporangium where pollen grains complete their development process. Anther development is particularly sensitive to biotic and abiotic stresses, which may lead to severe losses in yield^13^. *Ustilago maydis* causes disturbance in the anther lobes, and the infected organs increase in size compared with normal organs^14^. Millions of teliospores of *T. controversa* can develop in a grain of wheat^15^.

Plants have both inducible and performed mechanisms against pathogen infection^16,17^. The plant physical barriers, antimicrobial proteins and secondary metabolites (phytoanticipins) must be avoided or overcome for fungal pathogens to be able to invade a plant^18^. Once interactions between plants and pathogens are established, the elicitor molecules released and produced by fungal pathogens elicit further defenses, including the production of PR proteins, phytoalexins and the reinforcement of the cell walls^18–20^, ultimately leading to the hypersensitive response (HR), a type of programmed cell death that occurs at the site of pathogen attack^21^. Plant hormones are essential regulators of the different responses of plants to microbes. Plants release a blend of phytohormones, such as jasmonic acid (JA), salicylic acid (SA) and ethylene (ET), in response to fungal attack. The quantity, composition and timing of these phytohormones differ within plant species and based on the pathogen and its mode of infection^22^. Classically, the SA pathway delivers resistance against biotrophic pathogens, whereas the JA/ET pathways are associated with necrotrophic pathogens^23,24^.

Pathogenesis-related proteins are a group of functionally diverse inducible proteins that accumulate in plant tissue in response to pathogen attack^18^. These defense proteins are involved in active defense, potentially limiting pathogen development and spread^25^. Genes related to pathogenesis-related proteins accumulate within minutes to hours of pattern-triggered immunity (PTI) and effector-triggered immunity (ETI) induction, the expression of most of which is regulated by SA. To date, 17 different pathogenesis-related protein families (PR1-PR17) have been demonstrated in most plants^18,26^. In regard to their role in defense mechanisms, PR proteins have the ability to directly threaten pathogen integrity or release biochemicals by their enzymatic activity as elicitor molecules, which activates other plant defense-related pathways^27^. Most PR protein families include members whose activities are consistent with a role in plant defense against fungal/or oomycete pathogens: PR-1 (unknown), PR-2 (glucanases), PR-3, 4 (chitinases type I, II, IV, V, VI, VII), PR-4 (chitinases type I, II), PR-5 (thaumatin like proteins), PR-6 (proteinase-inhibitor), PR-7 (endoproteinase), PR-8 (chitinases III), PR-9 (peroxidase), PR-10 (ribonuclease like proteins), PR-11 (chitinase type 1), PR-12 (defensin), PR-13 (thionin), PR-14 (lipid transfer protein), PR-15 (oxalate oxidase), PR-16 (oxalate-oxidase like proteins) and PR-17 (unknown)^18,26^. Previous studies showed that PR-2, 3, 4, 8, and 11 act as antifungals. Similarly, PR-12^28,29^, PR-14^30^ and PR-5 and some members of PR-1 have been associated with activity against fungi and oomycetes. Additionally, the prominent PR-1 proteins are mostly used as markers of the enhanced defensive state conferred by pathogen-induced systemic acquired resistance (SAR), but their biological activity has remained elusive^31^. The overexpression or underexpression of these genes decreases or increases the disease intensity of various pathogens^32^. Callose-containing cell wall appositions, called papillae, are effective barriers that are induced at the site during the relatively early stages of pathogen invasion. Callose deposition in plants induces resistance against pathogens^33^, which is typically triggered by conserved pathogen-associated molecular patterns (PAMPs)^34^.

In this work, we tested the expression of defense-related genes (*PDF2.1, PDF2.4*, phenylalanine ammonia lyase (*PAL*), chitinase, ascorbate peroxidase (*APX*), and polyphenoloxidase (*PPO*)), PR genes (PR1a, PR2, PR5 and PR10), and PR-protein-encoding genes (chitinase 4, lipase, PR1.1, PR1.2, defensins, glucanases 2, lipid transfer protein-1 (LTP-1) and lipid transfer protein-2 (LTP-2) and callose deposition in resistant and susceptible wheat cultivars against *T. controversa* infection. The proliferation of fungal hyphae was also examined in the anther and ovary by using confocal microscopy. Additionally, the morpho-physiological attributes of wheat crops following *T. controversa* infection were characterized in both cultivars.

## Results

### Expression patterns of different regulators in wheat infected with *T. controversa*

**Figure 1:**
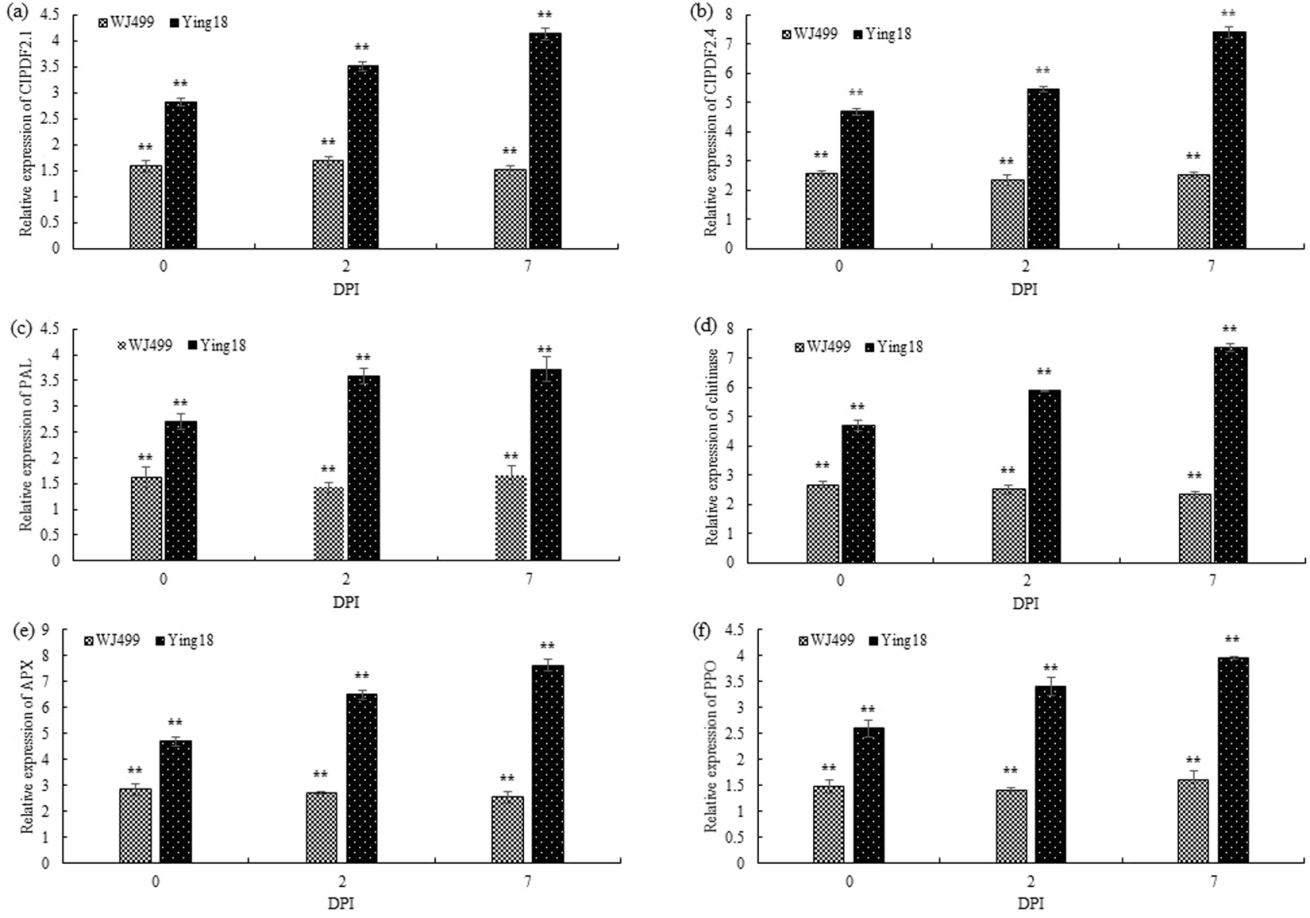
Quantitative real-time PCR (qRT-PCR) analysis of the transcription levels of pathogenesis-related genes in resistant (Ying18) and susceptible (WJ499) cultivars after *T. controversa* infection. (**a**) relative expression of CIPDF2.1 (**b**) relative expression of CIPDF2.4 (**c**) relative expression of phenylalanine ammonia lyase (*PAL*) (**d**) relative expression of chitinase (**e**) relative expression of ascorbate peroxidase (*APX*) (**f**) relative expression of polyphenoloxidase (*PPO*). The transcript abundances of the genes in the *T. controversa*-infected plants were relative to those of the control, and the significant differences were statistically analyzed based on three replications (Tukey’s test: **P < 0.01). Bars indicate the SEs.

The expression patterns of genes that activate various pathways, including *PDF2.1, PDF2.4*, *PAL*, chitinase, *APX*, and *PPO*, were investigated by qRT-PCR in both cultivars after *T. controversa* infection (Fig. 1). As shown in Fig. 1a, the relative expression of CIPDF2.1 was higher in the resistant cultivar than in the susceptible cultivar at 2 and 7 days post inoculation (DPI). The expression reached a peak at 7 DPI, an increase of 4.14-fold compared to the control. Similarly, in CIPDF2.4, the expression level gradually increased in the resistant cultivar over time from 2 to 7 DPI. The expression reached a peak at 7 DPI, increasing 7.39-fold compared to the control (Fig. 1b). The expression level continuously increased in the resistant cultivar, becoming 3.72-fold at 7 DPI in the resistant cultivar compared to control, but remaining almost the same in the susceptible cultivar (Fig. 1c). The expression level of chitinase was similar to that of *PAL*, although the expression level was highly induced at 7 DPI in the resistant cultivar (Fig. 1d). At 2 DPI, the relative expression of *APX* was 2.7 -fold and 6.49-fold in the susceptible and resistant cultivars when inoculated with the fungi, respectively, and the expression level continuously increased in the resistant cultivar, becoming 7.62-fold at 7 DPI in the resistant cultivar but slightly decreased in the susceptible cultivar (Fig. 1e). At 2 DPI, the relative expression of *PPO* was 1.41-fold and 3.4-fold in the susceptible and resistant cultivars when inoculated with the fungi, respectively, and continuously increased expression was noted in the resistant cultivar over time (Fig. 1f).

### Pathogenesis-related gene responses to exogenous hormone in wheat

**Figure 2:**
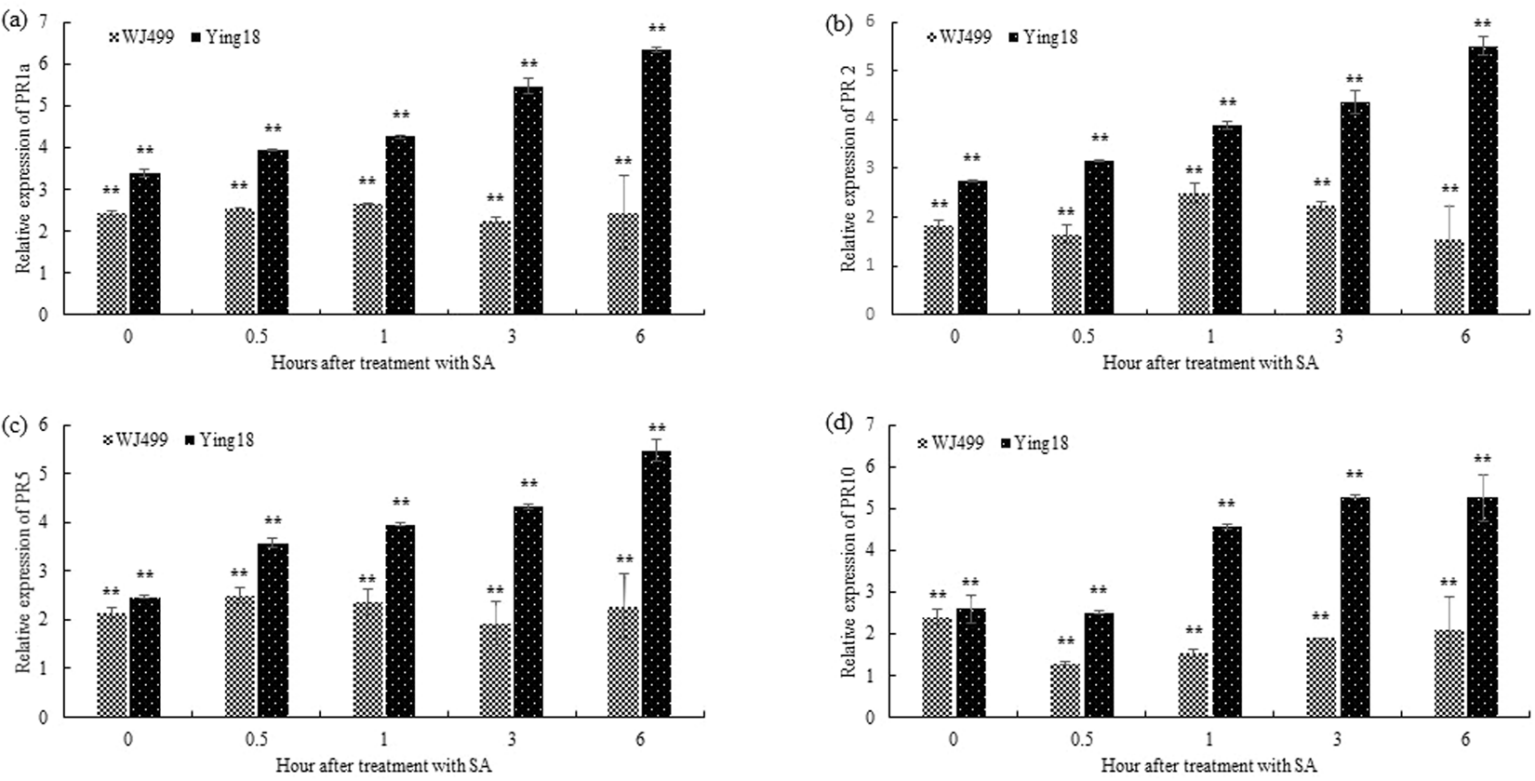
Quantitative real-time PCR (qRT-PCR) analysis of the transcription levels of pathogenesis-related genes in resistant (Ying18) and susceptible (WJ499) cultivars after salicylic acid (SA) treatment. (**a**) relative expression of PR1a (**b**) relative expression of PR2 (**c**) relative expression of PR5 (**d**) relative expression of PR10. The significant differences were statistically analyzed based on three replications (Tukey’s test: **P < 0.01). Bars indicate the SEs.

The transcriptional profiles of pathogenesis-related genes were analyzed by qRT-PCR in leaves of both cultivars after treatment with the exogenous defense-related hormone (SA). As shown in Fig. 2a, PR1a expression levels slowly increased in the resistant cultivar with time after SA treatment. The relative expression reached a peak at 6 hours after SA treatment and was induced 6.33-fold compared with that of control plants. However, the expression in the susceptible cultivar remained near that in the control plants. Similar responses were observed in PR2 and PR5 after SA treatments (Fig. 2b,c). The expression of PR10 was increased by SA treatment, but its expression level was similar at 3 and 6 hours after treatments (Fig. 2d).

### Expression patterns of pathogenesis-related genes in wheat after *T. controversa* infection

**Figure 3:**
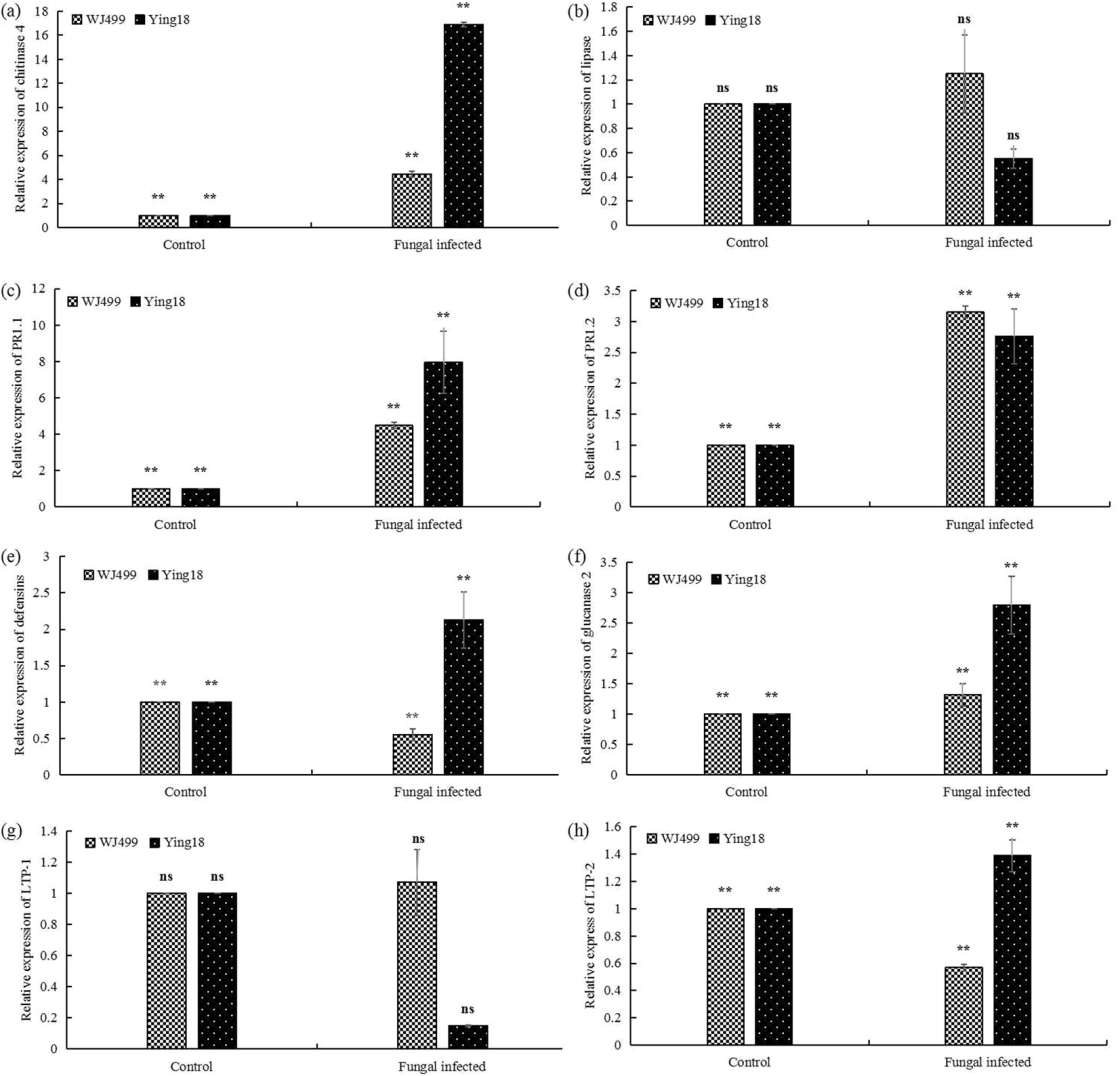
Quantitative real-time PCR (qRT-PCR) analysis of the transcription levels of pathogenesis-related genes in resistant (Ying18) and susceptible (WJ499) cultivars after *T. controversa* infection. (**a**) relative expression of chitinase 4 (**b**) relative expression of lipase (**c**) relative expression of PR1.1 (**d**) relative expression of PR1.2 (**e**) relative expression of defensins (**f**) relative expression of glucanase 2 (**g**) relative expression of LTP-1 (**h**) relative expression of LTP-2. The transcript abundances of the genes in the *T. controversa*-infected plants were relative to those of the control, and the significant differences were statistically analyzed based on three replications (Tukey’s test: *P < 0.05; **P < 0.01). ns = non-significant. Bars indicate the SEs.

Pathogenesis-related (PR) proteins, including chitinase 4, lipase, PR1.1, PR1.2, defensins, glucanases 2, lipid transfer protein-1 (LTP-1) and lipid transfer protein-2 (LTP-2), were shown to be involved in wheat resistance to fungal pathogens in previous publications^20^. To explore whether *T. controversa* regulates the expression of PR genes in resistant and susceptible wheat cultivars, the above-mentioned PR-protein-encoding genes were selected for transcriptional quantification by qRT-PCR. As shown in Fig. 3 acefh, qRT-PCR analysis revealed that the transcript abundances of chitinase 4, PR1.1, defensins, glucanases 2 and LTP-2 were enhanced in the resistant cultivar compared to the susceptible cultivar but that of PR1.2 was significantly decreased and those of lipase and LTP-1 were non-significantly decreased (Fig. 3d,b,g). The results clearly revealed that the expression of certain pathogenesis-related genes was higher in the resistant cultivar, which positively regulated resistance in the resistant cultivar.

### Determination of callose deposition in both cultivars against *T. controversa*

**Figure 4:**
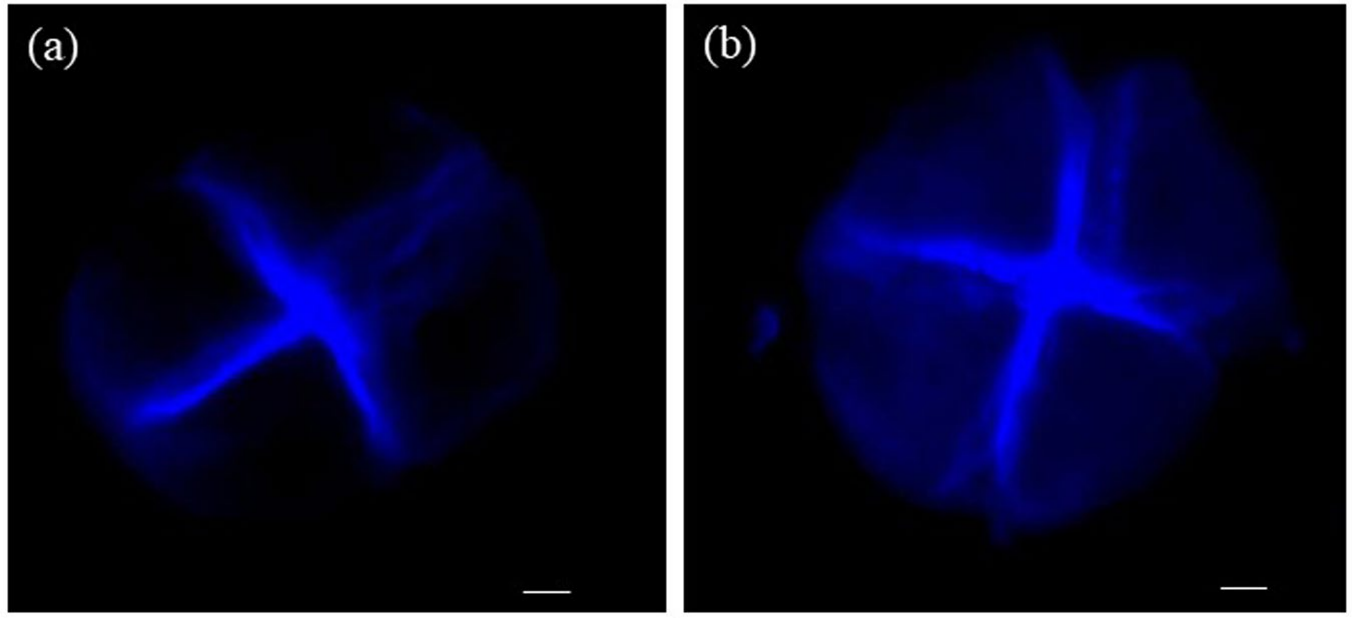
Callose deposition after *T. controversa* infection in anthers of resistant and susceptible cultivars at 10 DPI. (**a**) Callose deposition in the susceptible cultivar and (**b**) callose deposition in the resistant cultivar. Scale bars = 25 µm.

Callose deposition was investigated in the anthers of both cultivars after inoculated with *T. controversa*. The anthers were collected, stained with aniline blue, and examined by fluorescence microscopy. We consistently observed more callose deposits in the resistant cultivar (Fig. 4b) than in the susceptible cultivar (Fig. 4a).

### Proliferation of fungal hyphae in anther cells

**Figure 5:**
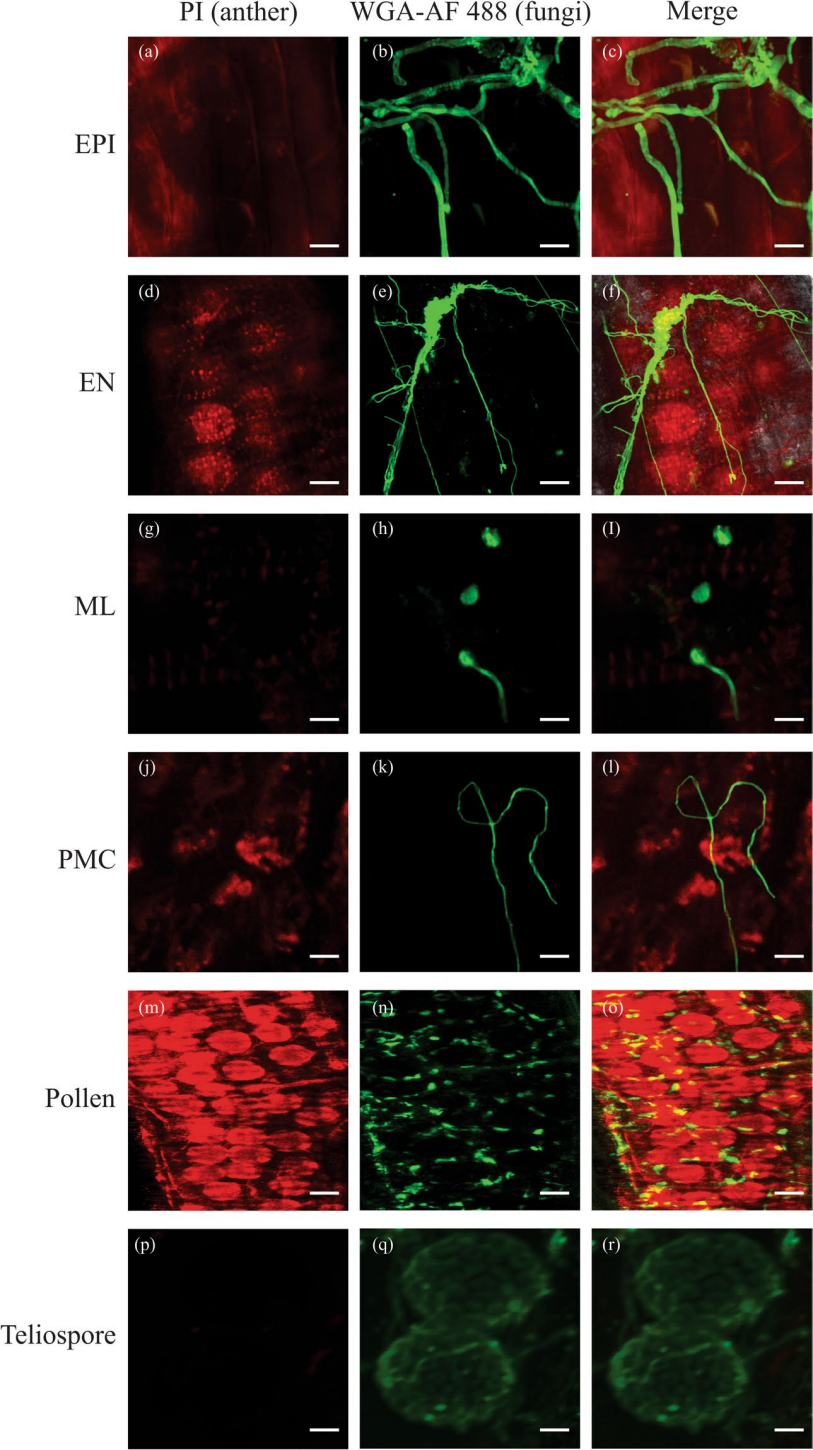
*T. controversa* location in anthers in the susceptible cultivar on 7 DPI. Propidium Iodide (PI) (red) stains the anther cell types, while Wheat Germ Agglutinin and Alexa Flour 488 conjugate (WGA-AF 488) (green) stains the fungal hyphae in the anthers. (**a–c**) Fungi located on EPI cell (**d–f**) hyphae located on EN cell (**g–i**) fungi located on ML cell (**j–l**) fungi located on PMC cell (**m–o**) fungi located on pollen cell (**p–r**) teliospores on epidermal cells. Scale bars = 25 µm (**a–c**, **g–r**), 50 µm (**d–f**). EPI, EN, ML, PMC, and pollen indicate the cell types during anther development.

**Figure 6:**
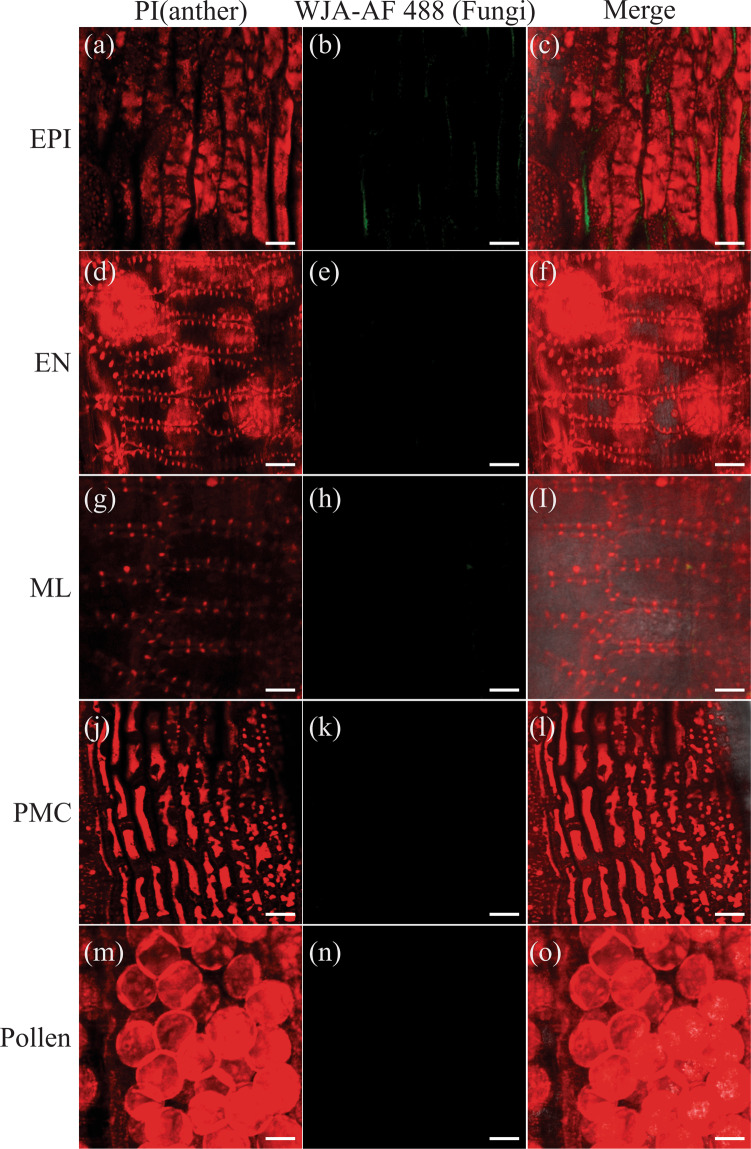
Examination of *T. controversa* in anthers in the resistant cultivar on 15 DPI. (**a–c**) Examination of fungi on EPI cells (**d–o**) Examination of fungi on EN cells (**d–f**), Examination of fungi on ML cells (**g–i**), Examination of fungi on PMCs (**j–l**), Examination of fungal pollen cells. Scale bars = 100 µm (**a–c**), 50 µm (**d–o**). EPI, EN, ML, PMC, and pollen indicate the cell types during anther development.

We examined the proliferation of *T. controversa* hyphae into anthers of both cultivars. The fungal penetration and colonization of anthers were examined by confocal microscopy. In general, we observed more proliferation of hyphae in anthers in the susceptible cultivar at 7 DPI than in the resistant cultivar (Fig. 5), and we could not find any hyphae in the anther cell types of the resistant cultivar until 10 DPI (Fig. 6).

### Proliferation of fungal hyphae into ovaries

**Figure 7:**
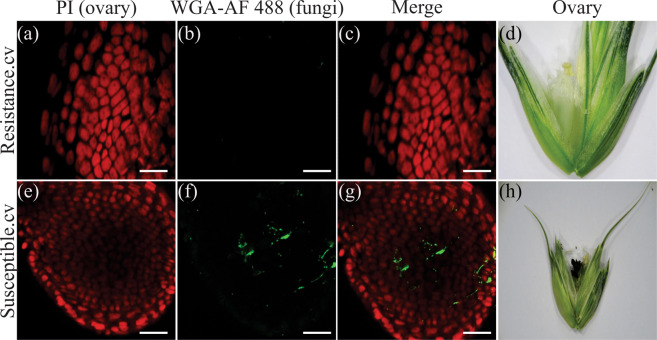
Examination of *T. controversa* in the ovaries of resistant (Ying18) and susceptible (WJ499) cultivars at 23 and 15 DPI, respectively. PI (red) stains the anther cell types, while WGA-AF 488 (green) stains the fungal hyphae in the ovary. (**a–c**) No fungal hyphae were seen on the cells of the ovaries in the resistant cultivar. (**d**) There were no symptoms on the ovaries of the resistant cultivar. (**e–g**) Location of fungi on ovary cells in the susceptible cultivar. (**h**) *T. controversa* converts the grain material into millions of teliospores. Scale bars = 50 µm.

We investigated the effect of *T. controversa* on ovaries in susceptible and resistant cultivars. No fungal hyphae were observed on the epidermal and sub-epidermal cells of ovary of the resistant cultivar after 23 DPI with confocal microscopy, and even the mature ovary was healthy (Fig. 7a–d). However, fungal hyphae were recorded on epidermal and sub-epidermal cells of the ovary in the susceptible cultivar after 15 DPI, and the infected ovary turned into black powder containing millions of teliospores (Fig. 7e–h).

### Anther length and width in inoculated and control plants

More than 300 anthers were collected from inoculated and control plants for length and width comparisons. The results showed that the inoculated plants had a significantly reduced anther length (Fig. S1a) and width (Fig. S1b) compared to those of control plants. In addition, plant height was measured at the ripening stage (Feekes stage 11), and the height of the plant from the ground to the tip of the spike and awns was used. The results showed that the plant height was reduced in *T. controversa*-infected plants in both cultivars compared to the control (Fig. S2).

### Evaluation of the dwarf bunt resistance

**Figure 8:**
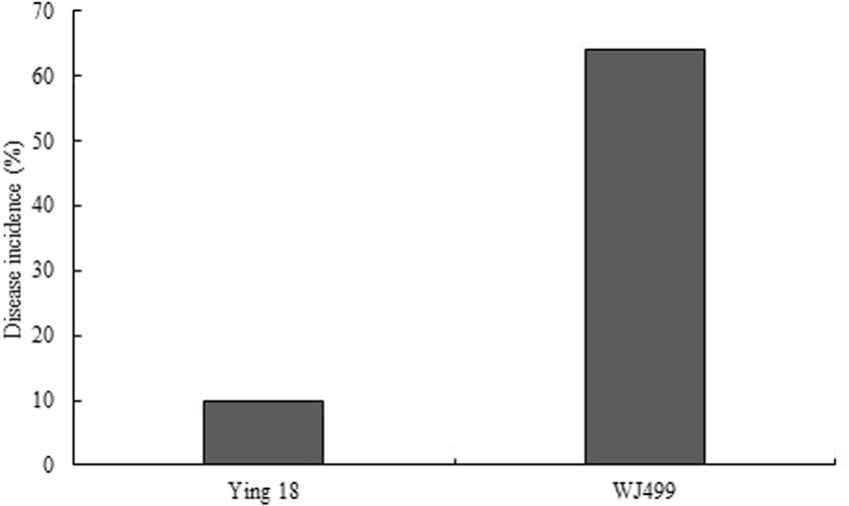
Level of disease incidence in resistant (Ying18) and susceptible (WJ499) cultivars to *T. controversa* infection. WJ499 showed 64% disease incidence, while Ying18 showed 10% disease incidence.

WJ499 as a susceptible cultivar used in this study, which had a high level of disease severity to dwarf bunt with 64%-infected heads (Fig. 8). This high level of infection in the susceptible cultivar confirmed that dwarf bunt infection was successful^35^.

## Discussion

Wheat is one of the major food crops worldwide. Here, we report the expression profiles of defense- and pathogenesis-related genes in resistant and susceptible wheat cultivars and the infection process of fungal hyphae in anthers and ovaries. The plant defensins genes (CIPDF2.1 and CIPDF2.4) are a major constituent of disease resistance, particularly against fungal pathogens^36^. Phenylalanine ammonia lyase have role to influenced the phenylpropanoid pathway because it catalyzes the conversion process of phenylalanine molecule to the cinnamic aid, which the is pioneer of lignin biosynthesis and salicylic acid molecule^37^. Chitinases are extensively accepted as playing major roles in plant defense mechanisms against fungal pathogens^18^. Ascorbate peroxidase is one of the active oxygen species hunting enzymes, and have key role in H_2_O_2_ metabolism of higher plants. Enough amount of endogenous ascorbate is very important for the maintenance of antioxidant system which protect the plants from oxidative damage from pathogen infections^38^. Polyphenols oxidized into quinones in the presence of polyphenol oxidase enzyme. The quinones are the antimicrobial compounds and have role in the lignification of plants cells during pathogen infection^39^. Our results showed that the expression of defense-related genes was higher in resistant than in susceptible cultivars at 2 and 7 DPI with *T. controversa* infection (Fig. 1). The differential pattern of defense-related genes shows the involvement of defensive pathways and their mechanistic importance in the response to *T. controversa*.

In this study, we also investigated the response of SA to the expression of pathogenesis-related genes, which showed that the expression of PR genes was higher in the resistant cultivar than in the susceptible cultivar (Fig. 2).

The expression of pathogenesis-related genes was induced upon infection with bunt pathogens^18,28,40–42^. Here, real-time PCR analysis results showed that infection with *T. controversa* triggers the transcriptional accumulation of pathogenesis-related genes more in resistant cultivar than in susceptible cultivar (Fig. 3). After infection of *T. controversa*, the transcriptional levels of chitinase 4, PR1.1, defensins, glucanases 2 and LTP-2 were higher in the resistant cultivar than in the susceptible cultivar (Fig. 3a,c,e,f,h). However, the expression of lipase, PR1.2 and LTP-1 was lower in the resistant cultivar than in the susceptible cultivar (Fig. 3b,d,g). The above results showed that some PR genes were activated upon infection by *T. controversa*. Previous studies showed that overexpression/silencing of these pathogenesis-related genes impaired or enhanced resistance to various diseases^32,38,43^. A common response by plants to fungal infection is the accumulation of callose, a (1,3)-β-glucan polymer, in the form of cell wall thickenings called papillae, at the site of pathogen infection^33^. The intensity and timing of pathogen-induced callose deposition depend on the pathogen and plant genetics. For example, plants that are subjected to fungal attack show systematic acquired resistance, which is linked with augmented levels of callose upon secondary pathogen infection^44^. In this work, the intensity of callose deposition was higher in resistant cultivar than in susceptible cultivar against *T. controversa* infection (Fig. 4).

The common, dwarf, and other related bunt fungi have a long infection and development process from seedling to grain filling^7,11,40^. In fact, the mycelium that develops in the infected seedling remains sparse until undergoing massive proliferation in floral tissue^45^. In the present study, we checked the varietal response to study the effects of *T. controversa* infection on anthers. Hyphae eventually proliferate into the pollen grains, and the release of these infected pollens for pollination is critical for normal plant reproduction. The produced seeds are transformed into a generally spherical sorus termed a bunt ball that contains a millions of teliospores^4^. The epidermal and sub-epidermal cells of ovaries were infected in the susceptible cultivar (Fig. 7). However, there were no fungal hyphae in the anther cells of the resistant cultivar, and the ovary cells were normal in the resistant cultivar (Figs. 6 and 7). Previous studies showed that dwarf bunt of wheat reduced plant height and other morphological attributes^46,47^, and we also confirmed these phenomena in our results.

## Materials and Methods

### Fungal material and culture

A fungal strain identified as *T. controversa* was provided by Blair Goates, United States Department of Agriculture (USDA), Agricultural Research Service (ARS), Aberdeen, Idaho, USA. Plates containing *T. controversa* teliospores on 2% soil-agar media were incubated under a 24 hours light cycle at 5 °C in an incubator (MLR 352 H, Panasonic, USA); after being covered with Parafilm for 60 days, teliospore germination and hyphal production were observed under an automated inverted fluorescence microscope (IX83, Olympus, Japan). Hyphae were collected and mixed with distilled water and used to inoculate wheat plants of both cultivars at a concentration of 10^6^ spores/ml with an OD_600_ of 0.15.

### Source of planting material and inoculation method

Two wheat (*Triticum aestivum* L.) cultivars i.e., Ying 18/lankao (Ying18) (resistant) and WJ499 (susceptible) were obtained from the Institute of Plant Protection (IPP), Chinese Academy of Agricultural Sciences (CAAS), Beijing, China. Both cultivars were tested in a greenhouse against *T. controversa* in 2017–2018, Ying18, which is known to be very resistant to *T. controversa* (with 7% infected heads), was used as the resistant cultivar in this work. WJ499, which was susceptible to *T. controversa* (52% infected heads), was used as a susceptible cultivar in this study. Seeds were surface sterilized with 30% NaClO for 1 min and then rinsed with sterile water 3 times and kept in plates with moist filter paper at 5 °C for one month to vernalize. After vernalization, seedlings were transplanted into pots filled with organic matter and soil at a ratio of 1:2% and grown in growth chambers (ARC-36, Percival, USA). Ten seedlings were transplanted in every pot. For each cultivar, 6 pots were used for inoculation, while 6 pots were used as controls. Wheat seedlings were grown at a 14 h light/10 hours dark cycle at 5 °C at the tillering stage and at 25 °C during the boot stage. At the early boot stage, the spikes were injected with 1 ml suspensions of *T. controversa* inoculum when the young wheat spikes were still wrapped by the leaf sheaths. Inoculation was repeated 3 times with a one-day interval. Plants injected with sterilized ddH_2_O were grown under the same conditions for use as the control treatment.

Both cultivars at the boot stage were sprayed with 1.0 mM SA and 0.1% Tween-20 solution. The plants treated with Tween solution were used as a control. The wheat leaves were sampled to detect the expression of defense-related genes.

### Callose deposition in anthers

The anthers were collected from both cultivars and dipped in absolute ethanol for 30 min. The application of absolute ethanol was repeated three times after 35 minutes until the tissue changed to white, and the anthers were crushed in solution using tweezers. One to two drops of 0.01% aniline blue staining solution were dropped onto glass slides, and the crushed anthers were incubated in the solution for 2 hours in the dark. The samples were observed under an inverted fluorescence microscope (Echo, USA) in DAPI mode. The excitation and emission wavelengths of aniline blue were 370 nm and 509 nm, respectively.

### RNA extraction and real-time PCR

To determine the expression levels of pathogenesis-related genes in the cultivars, samples were collected after 5 days of inoculation, and total RNA was extracted using a total RNA extraction kit (Solarbio, Beijing, China) according to the manufacturer’s instructions. The quantity and quality of RNA was quantified by using a Nano drop (Denovix, USA), and RNA was stored at −80 °C for further use. First-strand cDNA was synthesized by using 1.5 µg of purified total RNA, RT/RI enzyme and oligo (dT)18 Primer (TransGen) following the instructions of the kit (TransGen, Beijing, China) and stored at −20 °C for further use. Conventional PCR was performed in a 25-µl total reaction volume containing 12.5 µl of Master Mix, 1 µl of both primers, 2 µl of template and 8.5 µl of ddH_2_O for analyzing the efficiency of all primers. After PCR, gel electrophoresis was performed at 150 V for 30 min; the gel was stained with ethidium bromide, and the expected bands were visualized using the gel documentation system (ATTO, Korea). RNA extraction was performed with three biological replicates of inoculated and control plants of both cultivars. cDNA was synthesized from three biological replicates and four technical replicates for qRT-PCR analysis. Additionally, the same RNA extraction protocol was used for the analyzing the SA treatment and defense-related gene expression levels at different time intervals.

### Quantitative real-time PCR (qRT-PCR) of PR genes

qRT-PCR was performed using Top Green qPCR SuperMix in a volume of 20 µL according to the manufacturer’s instructions and the QuantStudio 5 real-time PCR system (Applied Biosystems, Beijing, China). The amplification of wheat actin was used as an internal control for normalizing all data. qRT-PCR reactions were set up with the following thermal cycles: pre-denaturation at 95 °C for 10 min and 40 cycles of 95 °C for 15 s, 58 °C for 30 s and 72 °C for 30 s. The 2 − ΔΔCt method^48^ was used to evaluate the relative expression of defense-related genes, where wheat actin was used as a reference. All primers for quantitative reverse transcription polymerase chain reaction (qRT-PCR) are listed in Table S1.

### Anther, ovary staining and confocal microscopy

The anthers were removed from each spikelet of both cultivars under a stereomicroscope (LeicaS6D, Germany). More than 200 anthers from inoculated and control plants of both cultivars were analyzed by confocal microscopy. Depending on the experimental conditions, the anthers were dipped in absolute ethanol for 30 min. Application of absolute ethanol was repeated three times after 35 min until the tissue changed to white. Hyphae in anthers were stained with the chitin-specific dye Wheat Germ Agglutinin and Alexa Flour 488 conjugate (WGA-AF488) (Invitrogen, Eugene, USA), and anther cells were stained with Propidium Iodide (PI) (Invitrogen, Eugene, USA)^49^. Samples were incubated at 25 °C for 60 min in 0.02% Tween 20 containing WGA-AF488 and PI at a ratio of 1:2. After staining, samples were rinsed 4–6 times in 1 × PBS (pH 7.4) (Suolaibao, Beijing, China) and stored in PBS at 4 °C without light. Fungal staining was performed at 25 °C. Samples were observed under a confocal laser scanning microscope (LeicaSP8, Germany) with excitation 448 nm/emission wavelengths of 510–550 nm (for WGA-AF 488) and excitation 561 nm/emission wavelengths of 570–730 nm (for PI). The same confocal microscopy method was used for ovary staining.

### Effect of *T. controversa* on physiological parameters

More than 300 anthers were collected from the control and inoculated plants for comparison. Anthers were dissected from florets using a stereomicroscope (S6D, Leica, Germany). Plant height were measured in both cultivars.

### Assessment of wheat plant resistance to *T. controversa*

Fifty heads of both cultivars were evaluated in response to *T. controversa* for disease assessment. Dwarf bunt resistance was scored as follows^47^:

$$\text{DB}\,=\,\text{number}\,\text{of}\,\text{infected}\,\text{heads}/\text{total}\,\text{number}\,\text{of}\,\text{heads}\times 100$$

The level of resistance was determined in both cultivars using the following scale:

$$\begin{array}{c}\text{Percentage}\,\text{of}\,\text{infected}\,\text{heads}\,0.0 \% =\text{highly}\,\text{resistant},\,1-5 \% =\text{resistant},\,5.1-10 \% \\ =\,\text{moderately}\,\text{resistant},\,10.1-30 \% =\text{moderately}\,\text{susceptible},\,30.1-50 \% \\ =\,\text{susceptible},\,50.1-100 \% =\text{highly}\,\text{susceptible}.\end{array}$$

### Statistical analysis

Data were statistically analyzed using one-way (ANOVA) followed by Tukey’s test in SPSS Statistics software. The results were considered significant at the 5% probability level (*P* ≤ 0.05). The standard errors were calculated in Excel 2016 (Microsoft).

## Supplementary information

Supplementary Table S1.

Supplementary Figure S1.

Supplementary Figure S2.
