# Supplementary material for: Characterization of the wheat cultivars against Tilletia controversa Kühn, causal agent of wheat dwarf bunt
Source: Sci Rep. 2020 Jun 3;10:9029. doi: 10.1038/s41598-020-65748-w (PMC7271121; doi:10.1038/s41598-020-65748-w)
Supplement: Supplementary file 1 — Supplementary Table S1. [file 41598_2020_65748_MOESM1_ESM.docx]

**Supplemental files**

Characterization of the wheat cultivars against *Tilletia controversa* Kühn, causal agent of wheat dwarf bunt

Ghulam Muhae-Ud-Din^1^, Delai Chen^1, 2^, Taiguo Liu^1^, Wanquan Chen^1*^ and Li Gao^1^*

**Table S1** Sequences of primers used in this study

| Primer Name | Sequence (5’-3’) | Usage |
| --- | --- | --- |
| Chitinase 4 F  Chitinase 4 R  Lipase F  Lipase R  PR 1.1 F  PR 1.1 R  PR 1.2 F  PR 1.2 R  Defensins F  Defensins R  Glucanase-2 F  Glucanase-2 R  LTP-1 F  LTP-1 R  LTP-2 F  LTP-2 R  ClPDF2.1 F  ClPDF2.1 R  ClPDF2.4 F  ClPDF2.4 R  PAL F  PAL R  Chitinase F  Chitinase R  APX F  APX R  PPO F  PPO R  PR1a F  PR1a R  PR2 F  PR2 R  PR5 F  PR5 R  PR10 F  PR10R  Actin F  Actin R | 5’-ttctggttctggatgaccaac-3’  5’-actgcttgcagtactccgtgt-3’  5’-cacaaaatatcgacccaccac-3’  5’-actgggtattcgtctgtcagc-3’  5’- actacgactacgggtccaaca-3’  5’- tcgtagttgcaggtgatgaag -3’  5’-cgtcttcatcacctgcaacta-3’  5’- caaacataaacacacgcacgta-3’  5’- tgtccaataagaactgcgcg -3’  5’- tggttccatgggctagctag-3’  5’-agcagaactggggactcttct-3’  5’-cacatacgtaccgcatacacg-3’  5’-acgtaggtactcctctcgctgt-3’  5’-gttgatcgaccacttcttctca-3’  5’-ggtcacacacacacacacaca-3’  5’-cgggagagaagtaacaaccaa-3’  5’-atgaagttcttttccgctgc-3’  5’-tcaaacgcagtgctttgtgcagaag-3’  5’atgaagtttctttttcagctgc-3’  5’-tcaaacgcagtgctttgtg-3’  5’- ccaatgttctgtccgtcctt-3’  5’- gagcttccctccaagatgtg -3’  5’-acggcgatatggttctggat-3’  5’-tagcgcttgtagaacccgat-3’  5’- tagggtcgtccgcga -3’  5’- ccccttacttgctcctc -3’  5’ gtccggacgaacaagaagaa-3’  5’- ggcgatgcttcacttggtat -3’  5’- cgtcttcatcacctgcaacta -3’  5’-caaacataaacacacgcacgta-3’  5’-ccgcacaagacacctcaagata-3’  5’-cgatgcccttggtttggtaga -3’  5’- acagctacgccaaggacgac -3’  5’-cgcgtcctaatctaagggcag -3’  5’-cgtggaggtaaacgatgag -3’  5’-gctaagtgtccggggtaat -3’  5’-ggaaaagtgcagagagacacg-3’  5’-tacagtgtctggatcggtggt-3’ | Q-RT-PCR  Q-RT-PCR  Q-RT-PCR  Q-RT-PCR  Q-RT-PCR  Q-RT-PCR  Q-RT-PCR  Q-RT-PCR  Q-RT-PCR  Q-RT-PCR  Q-RT-PCR  Q-RT-PCR  Q-RT-PCR  Q-RT-PCR  Q-RT-PCR  Q-RT-PCR  Q-RT-PCR  Q-RT-PCR  Q-RT-PCR |
