# Supplementary figures and images for: Characterization of the wheat cultivars against Tilletia controversa Kühn, causal agent of wheat dwarf bunt
Source: Sci Rep. 2020 Jun 3;10:9029. doi: 10.1038/s41598-020-65748-w (PMC7271121; doi:10.1038/s41598-020-65748-w)

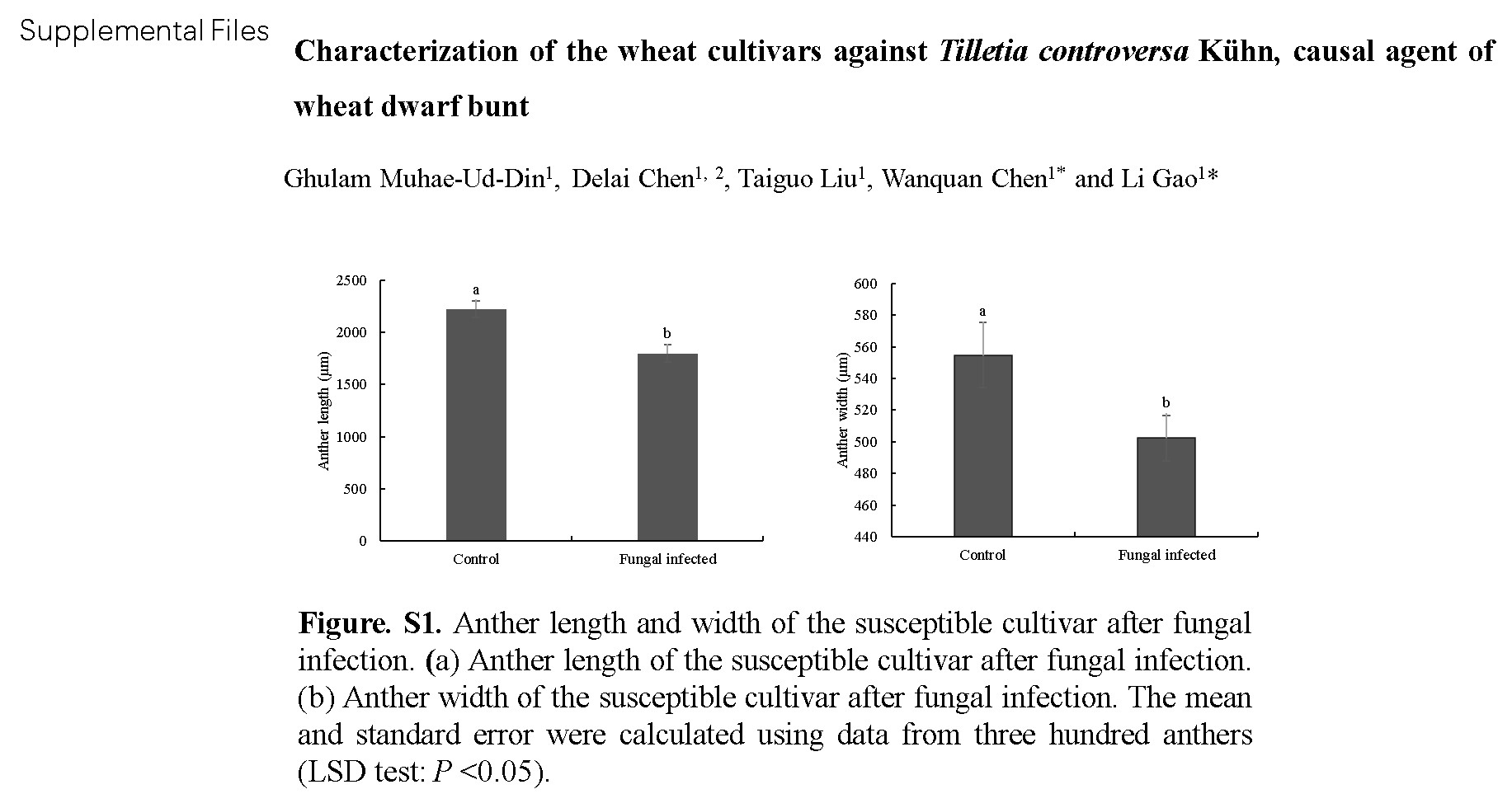

Supplement: Supplementary file 2 — Supplementary Figure S1. [file 41598_2020_65748_MOESM2_ESM.jpg]

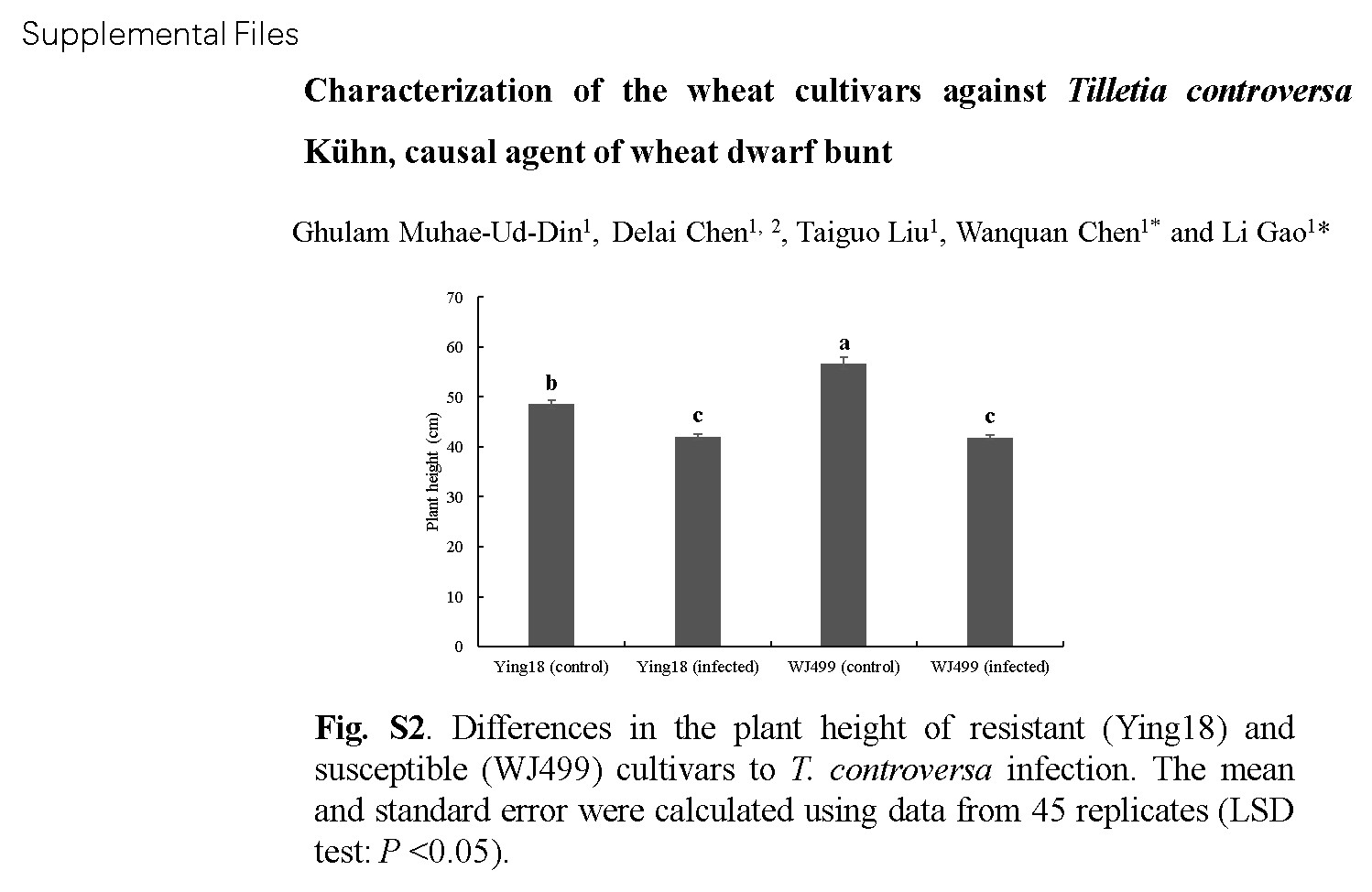

Supplement: Supplementary file 3 — Supplementary Figure S2. [file 41598_2020_65748_MOESM3_ESM.jpg]
